# Dietary Phytochemical Index and Its Relationship With Diminished Ovarian Reserve: Evidence From a Case–Control Study

**DOI:** 10.1002/edm2.70201

**Published:** 2026-06-23

**Authors:** Mahdieh Khodarahmi, Gholamreza Askari, Mahdi Vajdi, Amin Mokari‐Yamchi, Hatav Ghasemi‐Tehrani, Maryam Kalatehjari, Abed Ghavami

**Affiliations:** ^1^ Nutrition and Food Security Research Center Isfahan University of Medical Sciences Isfahan Iran; ^2^ Department of Community Nutrition, School of Nutrition and Food Science, Nutrition and Food Security Research Center Isfahan University of Medical Sciences Isfahan Iran; ^3^ Maternal and Childhood Obesity Research Center Urmia University of Medical Sciences Urmia Iran; ^4^ Department of Obstetrics and Gynecology School of Medicine, Isfahan University of Medical Sciences Isfahan Iran; ^5^ Obstetrics & Gynecology Department University of Washington Seattle USA; ^6^ Department of Clinical Nutrition, School of Nutrition and Food Sciences Isfahan University of Medical Sciences Isfahan Iran

**Keywords:** dietary phytochemical index, diminished ovarian reserve, infertility, ovarian function, phytochemicals

## Abstract

**Introduction:**

Diminished ovarian reserve (DOR) represents a significant contributor to female infertility and adverse reproductive outcomes. Although diet may play a role, the specific impact of phytochemical‐rich dietary patterns remains underexplored. So, we aimed to investigate the association between adherence to a dietary phytochemical index (DPI) and the likelihood of DOR among women attending fertility clinics.

**Methods:**

This case–control study enrolled 370 women, comprising 120 individuals diagnosed with DOR and 250 age‐ and body mass index (BMI)‐matched controls with normal ovarian reserve. A validated semi‐quantitative food frequency questionnaire (FFQ) was applied to assess dietary intakes and, accordingly, DPI was calculated as the proportion of total energy intake obtained from phytochemical‐abundant foods. Antral follicle count (AFC) and serum anti‐Müllerian hormone (AMH) measurements were utilized as indicators of ovarian reserve. The association between DPI and the odds of DOR was investigated using multivariable logistic regression models.

**Results:**

Our findings showed that higher DPI was associated with a reduced odds of DOR (Q4 vs. Q1 OR: 0.79; 95% CI: 0.55–0.93; *p*‐trend = 0.010). After adjustment for physical activity and energy intake, the association remained significant (OR: 0.80; 95% CI: 0.54–0.95; *p*‐trend = 0.033). In the fully adjusted model, which included additional adjustments for fat mass and body mass index, women in the highest DPI quartile had 27% lower odds of DOR compared to those in the lowest quartile (OR: 0.73; 95% CI: 0.42–0.97; *p*‐trend = 0.02). Besides, in the control group, AFC differed significantly across DPI quartiles (*p* < 0.001), with the highest mean in the second quartile.

**Conclusion:**

Our findings suggest that a phytochemical‐rich diet may help reduce the odds of DOR, highlighting the role of diet in reproductive health. However, further prospective studies and mechanistic research are warranted to confirm these results and clarify underlying pathways.

## Graphical abstract

A higher dietary phytochemical index was associated with lower odds of diminished ovarian reserve, highlighting the potential role of phytochemical‐rich diets in supporting ovarian function and reproductive health.

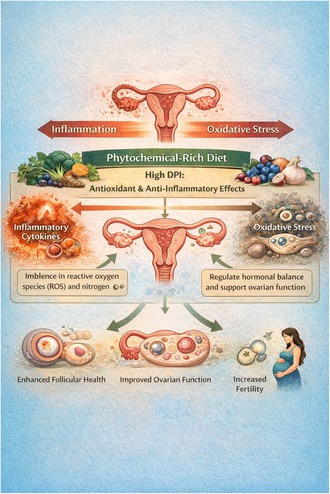

**Abbreviations**
AMH: anti‐Müllerian hormone
ART: assisted reproductive technology
BMI: body mass index
CI: confidence interval
DOR: diminished ovarian reserve
DPI: dietary phytochemical index
FFM: fat‐free mass
FFQ: food frequency questionnaire
FM: fat mass
HC: hip circumference
IL‐1β: interleukin‐β
IL‐6: interleukin‐6
IPAQ: international physical activity questionnaire
MAPK: mitogen‐activated protein kinase
MET: metabolic equivalent
NF‐κB: nuclear factor kappa B
OR: odds ratio
PCOS: polycystic ovary syndrome
ROS: reactive oxygen species
SD: standard deviation
TNF‐α: tumour necrosis factor‐alpha
WC: waist circumference
WHR: waist‐to‐hip ratio

## 1 Introduction

Ovarian reserve, which reflects the number and quality of the remaining ovarian follicles in a woman's ovaries, is a critical contributor to ovarian function [1]. Diminished ovarian reserve (DOR), which happens when this reserve declines to lower than expected for a woman's age, serves as a crucial predictor of fertility capacity, the ovarian response to assisted reproductive treatments, reproductive years, and the possible age at menopause [2]. Anti‐Müllerian hormone (AMH), secreted by granulosa cells of early antral follicles, and antral follicle count (AFC), assessed via transvaginal ultrasound, are currently recognised as the most promising quantitative markers of ovarian reserve [3]. Although ovarian reserve decreases with advancing age, considerable variations are observed among women of the same age group, which highlight significant interindividual differences [4]. These variabilities propose that beyond chronological age, additional determinants may influence the rate and degree of ovarian reserve decline [2].

Despite the well‐established role of advancing age in the determination of depletion of the ovarian oocyte pool, this process is also influenced by a complex interaction of factors including genetic predisposition, gynaecological conditions (e.g., endometriosis, tumours, infections, and prior ovarian surgery), environmental exposures (such as endocrine‐disrupting substances), and lifestyle‐related factors like psychological stress and, in particular, diet [4, 5]. Among these, diet has gained considerable attention due to its modulatory effects on oxidative stress, inflammation, and endocrine function, all of which play crucial roles in female reproductive health [6].

Considering nutritional factors, emerging evidence highlights the favourable effects of healthy dietary patterns such as the Mediterranean diet, characterised by high intake of vegetables and fruit, nuts, legumes, olive oil, fish, and whole grains, and low consumption of red and processed meats, sweets, and saturated fats, on fertility outcomes [7, 8]. The positive impact of these plant‐based diets may be attributed to their abundant content of essential nutrients and bioactive compounds such as vitamins, minerals, dietary fibre, and phytochemicals [9]. Phytochemicals are non‐nutritive bioactive substances, including polyphenols, carotenoids, phytosterols, and organosulfur compounds that are widely found in fruits, vegetables, nuts, whole grains, herbs and beverages such as tea and wine [10]. The likely reproductive health‐promoting effects of phytochemicals have been shown in previous epidemiological investigations across various populations [11, 12]. These natural compounds exert their benefits mainly by regulating oxidative stress, managing inflammation, and maintaining hormonal balance [13]. However, the majority of prior research on phytochemical intake has focused on individual nutrients, foods, or food groups [11, 14], while by comparison less attention has been given to comprehensive frameworks that assess the broader impact of phytochemical‐rich food combinations in the context of reproductive health and productivity.

Dietary Phytochemical Index (DPI) calculation provides a simple, cost‐effective approach to assess phytochemical intake and overall dietary quality [15]. Despite certain limitations, it could be applied in clinical practice [15]. DPI has attracted interest as a potentially modifiable factor in female reproductive health, supported by previous studies reporting inverse associations between DPI adherence and reproductive disorders such as polycystic ovary syndrome (PCOS) [16, 17]. Nonetheless, a significant knowledge gap remains regarding the specific association between this index and ovarian reserve status. As such, to shed light on this significant but neglected area, we aimed to investigate the potential relationship between DPI and the odds of having DOR in women attending fertility clinics.

## 2 Methods

### 2.1 Study Design and Participants

This research was conducted as a case–control study involving a total of 370 Iranian women, comprising 120 individuals diagnosed with DOR and 250 women with normal ovarian function serving as controls. We adhered to the STROBE (Strengthening the Reporting of Observational Studies in Epidemiology) guidelines in reporting this observational study. Participants were selected from infertility clinics associated with Isfahan University of Medical Sciences. Eligible women were between 18 and 45 years of age, with a body mass index (BMI) ranging from 20 to 35 kg/m^2^. Diagnosis of DOR was confirmed by two gynaecologists using criteria based on serum AMH levels of ≤ 0.7 ng/mL and/or an AFC of ≤ 4 [18]. To minimise misclassification and distinguish DOR from perimenopause or premature ovarian insufficiency (POI), women with clinical indicators suggestive of early ovarian decline such as prolonged or persistent menstrual irregularity and characteristic vasomotor symptoms (e.g., hot flashes and night sweats) were excluded from the study [19]. Frequency matching was applied to ensure similar distributions of important confounders between cases and controls. Specifically, cases and controls were matched based on age and BMI categories to minimise potential confounding. The required sample size for this study was estimated using standard formulas for unmatched case–control designs. Although cases and controls were matched on key characteristics in the study design, using the unmatched formula provides a conservative estimate of the required sample size, as matched designs typically increase statistical efficiency. Calculations were based on a two‐sided test, a 95% confidence level (α = 0.05), and 80% statistical power (β = 0.20). An anticipated odds ratio (OR) of 0.50 was assumed, reflecting a hypothesized 50% lower odds of DOR among women in the highest quartile of the DPI, based on previous evidence suggesting protective effects of phytochemical‐rich diets on reproductive health [17, 20]. The proportion of exposure (high DPI) among the control group was derived from our preliminary data, in which approximately 25% of control participants were categorised in the highest DPI quartile. The minimum required sample size was estimated at approximately 91 cases and 182 controls (273 participants in total). To account for an expected 10% non‐response or exclusion rate, the final target sample size was increased to 370 participants, including 120 women with DOR and 250 controls.

Individuals with a history of ovarian surgery, endometriosis, radiotherapy, chemotherapy, hormonal treatment, or special diets in the previous 3 months were excluded. Additionally, participants with endocrine or metabolic conditions were not eligible. Informed consent was obtained from all subjects, and the study protocol was approved by the Ethics Committee of Isfahan University of Medical Sciences (IR.ARI.MUI.REC.1401.297).

### 2.2 Dietary assessment and phytochemical index calculation

Dietary data were collected using a validated semi‐quantitative food frequency questionnaire (FFQ) containing 80 items [21]. Trained nutritionists conducted face‐to‐face interviews to complete the FFQs. Standard household measures were used to convert portion sizes into grams [22], and the Iranian‐adapted Nutritionist IV software was employed to analyse nutrient intake [23].

To better understand how phytochemical mixtures in the human diet contribute to health outcomes, researchers formulated the DPI, which represents the percentage of total daily calories consumed from foods rich in phytochemicals [24]. The DPI was calculated using the following formula [24]:

DPI = [daily energy obtained from foods rich in phytochemicals (kcal)/total daily energy intake (kcal) × 100].

Fruits, vegetables, legumes, whole grains, nuts, soy products, seeds, and olive oil were identified as primary sources of dietary phytochemicals and were therefore included in the DPI calculation. Foods such as potatoes, pickled vegetables, and vegetable powders were excluded due to their limited phytochemical content. Natural fruit juices were grouped with fruits, while vegetable juices and tomato‐based sauces were classified as vegetables, given their substantial phytochemical content, and all were incorporated into the DPI assessment. Following the calculation, DPI scores were divided into quartiles, with individuals in the highest quartile reflecting the greatest intake of phytochemical‐rich foods.

### 2.3 Anthropometric and Clinical Measures

Body weight and height were measured using calibrated instruments, and BMI was computed as weight in kilograms divided by height in meters squared. Waist circumference (WC) and hip circumference (HC) were measured to calculate the waist‐to‐hip ratio (WHR). Fat mass (FM) and fat‐free mass (FFM) were assessed using bioelectrical impedance analysis (InBody 770). Systolic and diastolic blood pressures were recorded using an automated digital monitor after participants rested for 15 min. Physical activity levels were assessed with the validated Iranian version of the International Physical Activity Questionnaire (IPAQ), reported in metabolic equivalent hours per day (MET‐h/day) [25]. Serum AMH levels were measured using ELISA kits (Monobind, California, USA), according to the manufacturer protocols, and AFC was evaluated using transvaginal ultrasonography on the third day of a spontaneous menstrual cycle.

### 2.4 Statistical Analysis

Continuous variables in this study were presented as means and standard deviations (SD), and differences between groups were analysed using the independent sample *t*‐test and one‐way ANOVA. Categorical data were summarised as frequencies (%), and the chi‐square test was used to assess differences between the case and control groups. Logistic regression analysis was conducted to estimate the ORs and 95% confidence intervals (CIs) for DOR across DPI quartiles. Model 1 was adjusted for physical activity and calorie intake; Model 2 additionally adjusted for BMI and fat mass. All analyses were conducted using SPSS version 22 (IBM Corp, Armonk, NY, USA). Statistical significance was determined for *p*‐values below 0.05.

## 3 Results

**FIGURE 1:**
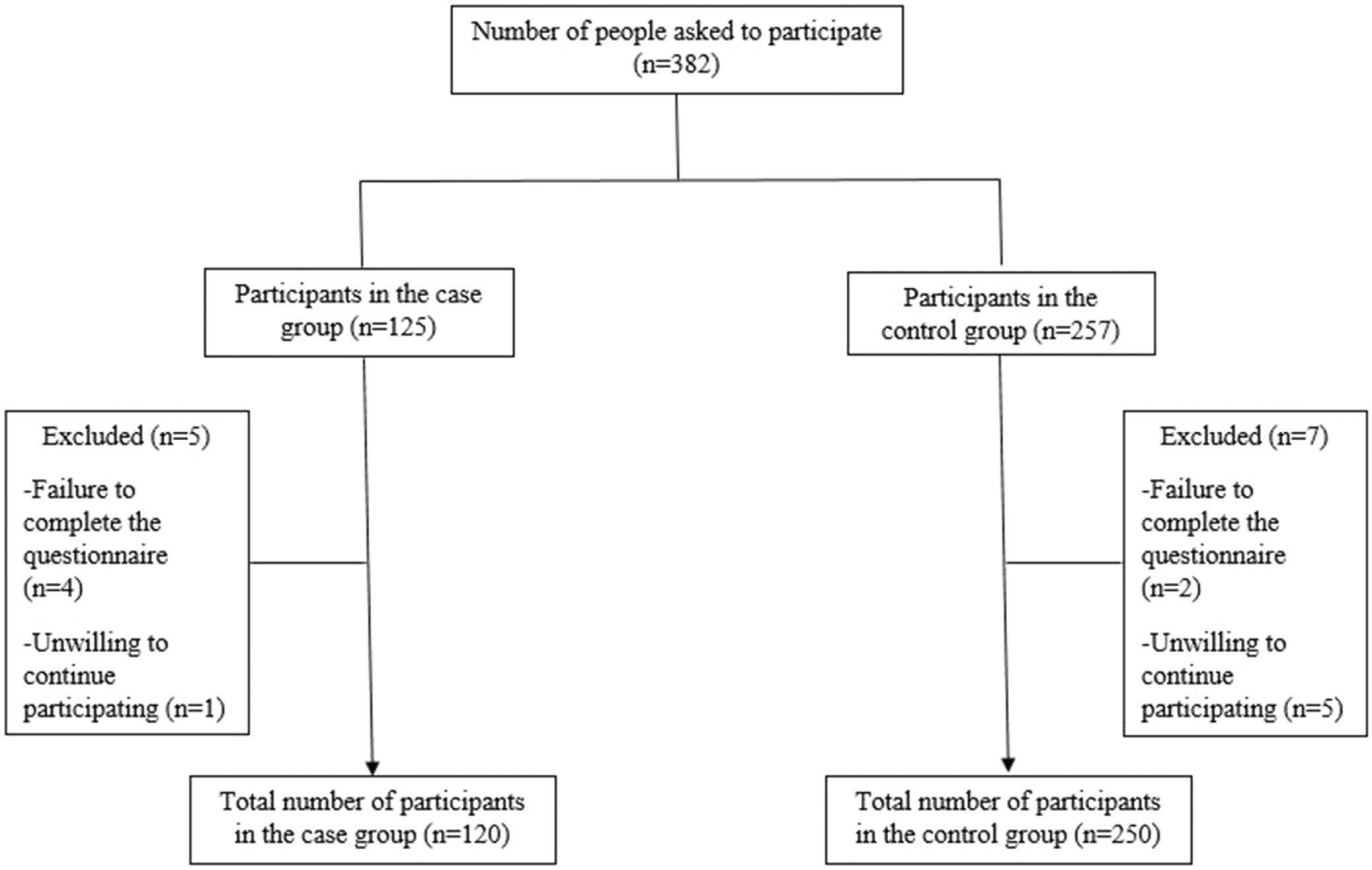
Flowchart of participant selection and study enrollment. Participants were 4 matched based on age and BMI at the time of recruitment to control for baseline confounders.

In this study, 120 women diagnosed with DOR were recruited as the case group, and 250 age‐ and BMI‐matched women with normal ovarian reserve were enrolled as the control group. A total of 382 women were initially recruited. Nonetheless, 12 participants were excluded from the study: 6 due to unwillingness to participate and 6 due to incomplete questionnaire responses (Figure 1). The baseline characteristics of participants are presented in Table 1. Women with DOR had significantly higher FM (38.47 vs. 36.47 kg, *p* = 0.020), WC (102.23 vs. 91.70 cm, *p* = 0.002), and WHR (0.90 vs. 0.86, *p* = 0.003), and significantly lower AFC (2.34 vs. 9.59, *p* < 0.001) and AMH levels (0.56 vs. 4.11 ng/mL, p < 0.001), compared to controls.

**TABLE 1:** Baseline characteristic of study participants.

Variable		Case (*N* = 120)	Control (*N* = 250)	*p* ^a^
Age (years)		33.37 ± 3.24	32.91 ± 3.15	0.196
BMI (kg/m^2^)		29.85 ± 2.49	27.75 ± 3.45	0.235
Weight (kg)		80.96 ± 4.78	79.26 ± 8.41	0.487
FM (kg)		38.47 ± 7.05	36.47 ± 8.91	0.020
FFM (kg)		57.99 ± 11.33	60.12 ± 11.97	0.098
WC (cm)		102.23 ± 35.95	91.70 ± 12.43	0.002
HC (cm)		109.10 ± 31.59	106.10 ± 11.57	0.316
WHR		0.90 ± 0.12	0.86 ± 0.08	0.003
SBP (mmHg)		122.18 ± 12.77	123.58 ± 14.03	0.341
DBP (mmHg)		79.41 ± 11.67	81.85 ± 10.48	0.056
Physical activity (MET/h/day)		19.05 ± 4.12	18.98 ± 4.51	0.896
Socioeconomic status (SES) (%)	Low	10 (8.3)	19 (7.6)	0.252
	Middle	50 (41.7)	127 (50.8)	
	High	60 (50)	104 (41.6)	
Education (%)	Illiterate	14 (11.7)	34 (13.6)	< 0.001
	≤ High school/diploma	31(25.8)	121 (48.4)	
	≥ College degree	75 (62.5)	95 (38)	
Occupation (%)	Housewife	82 (68.3)	184 (73.6)	< 0.001
	Employed	26 (21.7)	10 (4)	
	Student	12 (10)	56 (22.4)	
Pervious pregnancy	Yes	99 (82.5)	203 (81.2)	0.441
	No	21 (17.5)	47 (18.8)	
AFC count		2.34 ± 1.19	9.59 ± 2.24	< 0.001
AMH (ng/ml)		0.56 ± 0.71	4.11 ± 1.18	< 0.001

*Note:* Quantitative variables are expressed as mean ± SD and qualitative variables expressed as *n* (%). The SES scored was evaluated based on the education level of both subjects and the family head, job of both subjects and the family head, family size, home status, and home type by using a self‐reported questionnaire.

Abbreviations: AFC; antral follicle count, BMI; body mass index, DBP; diastolic blood pressure, DOR; duration, diminished or decreased ovarian reserve, FFM; fat‐free mass, FM; fat mass, HC; hip circumference, SBP; systolic blood pressure, WC; waist circumference, WHR; waist‐to‐hip ratio.

^a^ *p* values resulted from independent *t*‐tests for quantitative and Chi‐square for qualitative variables between the two groups.

Table 2 summarises the demographic, anthropometric, and clinical characteristics of participants across quartiles of the DPI, separately for case and control groups. No statistically significant differences were found across DPI quartiles with respect to age, BMI, WC, fat mass, or fat‐free mass (*p* > 0.05) in either the case or control group. However, among the control group, the AFC differed significantly across DPI quartiles (*p* < 0.001), with the highest mean count observed in the second quartile.

**TABLE 2:** Characteristic of study participants according to quartiles of DPI.

Variable		Case (120)					Control (250)				
		Q1	Q2	Q3	Q4	*p* *	Q1	Q2	Q3	Q4	*p* *
		< 17.8	17.8–22.7	22.8–28.3	> 28.4		< 19.2	19.3–25.8	25.9–29.2	> 29.3	
Age (years)		34.00 ± 3.45	33.06 ± 2.98	32.96 ± 3.32	33.59 ± 3.27	0.583	33.37 ± 2.95	33.33 ± 2.98	32.83 ± 3.51	32.17 ± 3.06	0.101
BMI (kg/m^2^)		29.65 ± 2.51	30.60 ± 2.47	29.71 ± 2.80	29.36 ± 2.08	0.248	29.65 ± 2.51	30.60 ± 2.47	29.71 ± 2.80	29.36 ± 2.08	0.396
Weight (kg)		81.48 ± 3.29	83.09 ± 5.02	83.10 ± 3.64	81.64 ± 4.11	0.236	81.48 ± 3.29	83.09 ± 5.02	83.10 ± 3.64	81.64 ± 4 11	0.155
FM (kg)		37.40 ± 6.81	36.62 ± 5.37	40.96 ± 9.15	39.04 ± 5.73	0.078	37.40 ± 6.81	36.62 ± 5.37	40.96 ± 9.15	39.04 ± 5.73	0.993
FFM (kg)		55.98 ± 11.80	60.43 ± 11.96	56.96 ± 10.61	58.62 ± 10.87	0.436	58.72 ± 12.23	62.69 ± 12.65	58.92 ± 11.33	60.18 ± 11.52	0.229
WC (cm)		101.19 ± 31.27	89.83 ± 26.56	113.13 ± 40.37	105.42 ± 41.90	0.081	91.29 ± 11.64	89.48 ± 12.55	92.73 ± 11.99	93.26 ± 1 3.42	0.326
HC (cm)		110.64 ± 35.60	97.95 ± 12.53	119.80 ± 38.40	108.26 ± 30.97	0.058	105.05 ± 11.31	104.29 ± 11.75	106.60 ± 9.71	108.42 ± 13.11	0.196
WHR		0.94 ± 0.10	0.87 ± 0.10	0.90 ± 0.16	0.88 ± 0.09	0.173	0.87 ± 0.08	0.86 ± 0.09	0.86 ± 0.07	0.86 ± 0.07	0.827
SBP (mmHg)		118.22 ± 11.43	122.74 ± 13.38	122.5 ± 12.84	125.60 ± 12.91	0.167	123.14 ± 14.37	123.79 ± 13.86	125.79 ± 14.67	121.58 ± 13.19	0.408
DBP (mmHg)		77.25 ± 11.39	79.32 ± 12.92	81.06 ± 11.07	80.14 ± 11.41	0.627	81.20 ± 10.92	82.32 ± 11.43	83.09 ± 10.60	80.79 ± 10.51	0.626
AFC count		1.93 ± 1.28	2.48 ± 1.36	2.53 ± 0.73	2.42 ± 1.25	0.179	9.45 ± 1.98	10.67 ± 2.64	8.80 ± 1.49	9.44 ± 2.31	< 0.001
AMH (ng/ml)		0.52 ± 0.25	0.50 ± 0.19	0.68 ± 0.39	0.54 ± 0.17	0.551	4.22 ± 1.19	4.00 ± 1.24	4.04 ± 1.26	4.18 ± 1.04	0.683
Physical activity (MET/h/day)		19.03 ± 4.30	20.51 ± 3.48	18.06 ± 4.46	18.50 ± 3.94	0.760	19.09 ± 4.55	18.61 ± 4.53	19.49 ± 4.42	18.74 ± 4.59	0.104
Socioeconomic status (SES) (%)	Low	3 (9.7)	3 (9.7)	3 (10.0)	1 (3.6)	0.511	5 (8.1)	4 (6.5)	3 (4.8)	7 (11.1)	0.670
	Middle	9 (20.0)	13 (41.9)	16 (50)	9 (33.3)		36 (58.1)	31 (50.0)	31 (49.2)	29 (46.0)	
	High	19 (61.3)	15 (48.4)	15 (50.0)	11 (39.3)		21 (33.9)	27 (43.5)	29 (46.0)	27 (42.9)	
Education (%)	Illiterate	5 (16.1)	0 (0.0)	7 (23.3)	2 (7.1)	0.032	7 (11.3)	6 (9.7)	9 (14.3)	12 (19.0)	0.206
	≤ High school/diploma	4 (12.9)	8 (25.8)	9 (30.0)	10 (35.7)		27 (43.5)	29 (46.8)	29 (46.0)	36 (57.1)	
	≥ College degree	22(71.0)	23 (74.2)	14 (46.7)	16 (57.1)		28 (45.2)	27 (43.5)	25 (39.7)	15 (23.8)	
Occupation	Housewife	21 (67.7)	21 (67.7)	22 (73.7)	18 (64.3)	0.807	44 (71.0)	48 (77.4)	46 (73.0)	46 (73.0)	0.959
	Employed	5 (16.1)	7 (22.6)	6 (20.0)	8 (28.6)		3 (4.8)	1 (1.8)	3 (4.8)	3 (4.8)	
	Student	5 (16.1)	3 (9.7)	2 (6.7)	2 (7.1)		12 (17.9)	15 (26.8)	16 (27.1)	13 (19.1)	
Pervious Pregnancy	No	26 (83.9)	28 (90.3)	24 (80.0)	21 (75.0)	0.462	51 (82.3)	48 (77.4)	56 (88.9)	48 (76.2)	0.251
	Yes	5 (16.1)	3 (9.7)	6 (20.0)	7 (25.0)		11 (17.7)	14 (22.6)	7 (11.1)	15 (23.8)	

*Note:* Quantitative variables are expressed as mean ± SD and qualitative variables expressed as *n* (%). The SES score was evaluated based on the education level of both subjects and the family head, job of both subjects and the family head, family size, home status, and home type by using a self‐reported questionnaire.

Abbreviations: AFC; antral follicle count, BMI; body mass index, DBP; diastolic blood pressure, DOR; diminished ovarian reserve, DPI; dietary phytochemical index, FFM; fat‐free mass, FM; fat mass, HC; hip circumference, SBP; systolic blood pressure, WC; waist circumference, WHR; waist‐to‐hip ratio.

* *p* values resulted from independent *t*‐tests for quantitative and Chi‐square for qualitative variables between the two groups.

Table 3 presents dietary intake profiles across DPI quartiles. As expected, higher DPI scores were consistently associated with healthier dietary patterns in both groups. Among controls, total energy, protein, carbohydrate, and fat intakes increased significantly across DPI quartiles (*p* < 0.001). Intake of phytochemical‐rich foods such as fruits, vegetables, legumes, nuts, whole grains, olives, soy, tea, and spices showed a clear increasing trend with higher DPI scores (all *p* < 0.001). Participants in the highest DPI quartile consumed significantly more fruits (589 ± 425 g/day), vegetables (648 ± 402 g/day), legumes, whole grains, soy, and tea compared to those in the lowest quartile (all *p* < 0.001). Saturated fat intake remained relatively stable in controls. However, among patients with DOR, those assigned to the highest quartile had a higher consumption of SFA in comparison with the lowest category (*p* < 0.001).

**TABLE 3:** Energy, nutrient and phytochemical‐rich foods intake across quartiles of the DPI in case and control groups.

Variable	Case (120)					Control (250)				
	Q1	Q2	Q3	Q4	*p* *	Q1	Q2	Q3	Q4	*p* *
	< 17.8	17.8–22.7	22.8–28.3	> 28.4		< 19.2	19.3–25.8	25.9–29.2	> 29.3	
Energy (Kcal/day)	2452.94 ± 740.01	2394.31 ± 627.33	2617.39 ± 824.12	2870.34 ± 887.06	< 0.001	2375.32 ± 542.68	2483.23 ± 602.37	2583.63 ± 702.31	2883.92 ± 702.21	< 0.001
Protein (g/day)	81.9 ± 31.94	79.1 ± 29.89	90.8 ± 29.51	96.8 ± 28.24	< 0.001	94.95 ± 29.51	98.61 ± 31.27	99.24 ± 34.42	104.84 ± 33.82	< 0.001
Carbohydrate (g/day)	328.36 ± 128.11	340.87 ± 128.57	385.36 ± 140.79	398.21 ± 144.62	< 0.001	342.12 ± 103.52	378.65 ± 115.42	394.89 ± 113.17	425.27 ± 118.19	< 0.001
Total fibre (g/day)	18.63 ± 11.71	17.92 ± 8.38	18.31 ± 8.90	20.17 ± 13.86	0.257	22.64 ± 10.25	23.35 ± 9.96	24.25 ± 9.20	24.34 ± 10.61	0.632
Fat (g/day)	78.33 ± 32.27	79.66 ± 22.81	80.42 ± 36.68	86.72 ± 30.85	< 0.001	85.08 ± 31.10	88.79 ± 32.68	90.21 ± 32.10	94.24 ± 33.71	< 0.001
SFA (g/day)	27.11 ± 14.18	22.82 ± 11.34	29.63 ± 11.74	32.87 ± 12.78	< 0.001	30.21 ± 12.43	29.93 ± 11.24	29.51 ± 11.32	30.98 ± 12.39	0.217
PUFA (g/day)	18.2 ± 8.33	15.62 ± 8.12	19.21 ± 6.34	21.59 ± 7.29	< 0.001	23.10 ± 6.92	23.16 ± 7.29	25.19 ± 8.10	26.53 ± 8.91	0.741
DPI component										
Fruits (g/day)	222.36 ± 155.12	285.01 ± 209.66	378.33 ± 309.14	589 ± 425.33	< 0.001	363.3 ± 27.4	423.75 ± 22.12	436.43 ± 12.27	517.2**3** ± 14.58	< 0.001
Vegetables (g/day)	331.35 ± 181.87	276.24 ± 179.77	432.39 ± 283.76	648.36 ± 402.08	< 0.001	345.23 ± 7.5	355.45 ± 10.42	362.35 ± 8.61	379.46 ± 9.86	< 0.001
Legumes (g/day)	28.82 ± 3.12	22.35 ± 2.37	35.6 ± 4.27	54.6 ± 5.83	< 0.001	48.21 ± 4.15	55.61 ± 5.34	56.95 ± 5.17	60.74 ± 6.29	< 0.001
Nuts (g/day)	10.47 ± 2.58	13.72 ± 2.82	20.1 ± 5.13	26.8 ± 4.28	< 0.001	13.39 ± 1.35	12.92 ± 1.65	14.19 ± 1.45	22.21 ± 1.74	< 0.001
Whole grain (g/day)	230 ± 90.6	352.23 ± 121.37	440.28 ± 152.42	668.19 ± 159.34	< 0.001	248.59 ± 85.83	255.8 ± 89.26	256.63 ± 92.12	344.91 ± 120.47	< 0.001
Olive (g)	6.5 ± 2.58	8.72 ± 2.82	12.1 ± 3.21	16.8 ± 4.28	< 0.001	2.5 ± 2.01	3.8 ± 2.82	3.9 ± 2.13	5.8 ± 3.28	< 0.001
Tea (g)	225.35 ± 150.87	350.24 ± 190.77	398.39 ± 283.76	480.36 ± 321.08	< 0.001	290.35 ± 190.87	310.29 ± 202.79	385.39 ± 294.76	390.36 ± 321.08	< 0.001
Soy (g)	1.12 ± 1.58	1.5 ± 1.13	1.9 ± 1.7	2.5 ± 1.54	< 0.001	1.25 ± 1.41	1.87 ± 1.24	1.91 ± 1.05	2.02 ± 1.80	< 0.001
Spices (g)	6.26 ± 2.47	7.34 ± 2.62	8.76 ± 2.37	9.81 ± 2.25	< 0.001	5.62 ± 2.14	6.14 ± 2.38	7.27 ± 2.31	9.71 ± 3.17	< 0.001
Caffeine (mg)	1428.33 ± 1374.32	1322.98 ± 1035.47	1517.33 ± 1421.52	1675.14 ± 1452.27	0.673	1366.18 ± 1171.28	1453.37 ± 1151.16	1474.2 ± 1153.56	1597.5 ± 1185.33	0.547
Coffee (mg)	262.19 ± 128.38	282.31 ± 112.37	287.22 ± 125.37	396.11 ± 123.47	0.254	210.5 ± 124.61	274.5 ± 123.35	269.6 ± 119.42	353.10 ± 134.83	0.324

*Note:* Variables are expressed as mean ± SD.

Abbreviations: DPI; dietary phytochemical index, SFA; saturated fatty acids, PUFA; polyunsaturated fatty acids.

* *p* values resulted from ANOVA test.

Logistic regression results are shown in Table 4. In the crude model, higher DPI was associated with a reduced odds of DOR (Q4 vs. Q1 OR: 0.79; 95% CI: 0.55–0.93; *p*‐trend = 0.01). After adjustment for physical activity and energy intake (Model 1), the association remained significant (OR: 0.80; 95% CI: 0.54–0.95; *p*‐trend = 0.033). In the fully adjusted model (Model 2), women in the highest DPI quartile had 27% lower odds of DOR compared to those in the lowest quartile (OR: 0.73; 95% CI: 0.42–0.97; *p*‐trend = 0.02).

**TABLE 4:** Crude and adjusted associations between DPI scores and the odds of DOR among the study population.

DPI (Range)	Q1	Q2	Q3	Q4	*p*
	(< 17.8)	(17.8–22.7)	(22.8–28.3)	(> 28.4)	
DOR/Control	31/62	31/62	30/63	28/63	
Crude	Ref (1.00)	0.88 (0.64–1.09)	0.85 (0.63–1.07)	0.79 (0.55–0.93)	0.01
Model 1	Ref (1.00)	0.90 (0.66–1.14)	0.93 (0.64–1.22)	0.80 (0.54–0.95)	0.033
Model 2	Ref (1.00)	0.81 (0.54–1.19)	0.94 (0.66–1.31)	0.73 (0.42–0.97)	0.02

*Note:* Multivariable logistic regression models were used with adjustment of potential confounders. Model 1: Physical Activity + Energy intake (kcal/day). Model 2: Model 1 + Fat Mass (kg) and body mass index. Participants were matched based on age and BMI at the time of recruitment to control for baseline confounders.

Abbreviations: DOR; diminished ovarian reserve, DPI; dietary phytochemical index.

## 4 Discussion

This study is, to our knowledge, the first to explore the relationship between the DPI and DOR in women of reproductive age. Our findings suggest that individuals with higher DPI scores, reflecting greater intake of phytochemical‐rich foods, had significantly lower odds of experiencing DOR. In particular, women in the highest DPI quartile had 27% lower odds of DOR compared to those in the lowest quartile, even after accounting for potential confounding factors such as physical activity, energy intake, BMI, and fat mass. These findings indicate that diets rich in phytochemicals may exert a protective effect against ovarian aging and support reproductive health. Moreover, among the control group, significant variation in AFC was observed across DPI quartiles, further supporting the link between dietary quality and ovarian reserve.

DOR, defined by a decline in both the number and quality of oocytes, may result in early onset of menopause and reduced fertility potential [26]. This decline typically represents a physiological consequence of advancing age, occurring in the absence of underlying pathological conditions [26]. However, the variable rate of ovarian reserve decline among reproductive‐aged women suggests influences beyond age [2]. Although the exact aetiology of DOR is not fully understood, several factors have been implicated, including ovarian surgery, exposure to gonadotoxic treatments, genetic predispositions, autoimmune disorders, and various environmental or lifestyle factors such as smoking, inadequate nutrition, and chronic psychological stress [2, 27]. Despite the lack of comprehensive studies examining the specific effects of diet on ovarian reserve, available evidence suggests that vegetarian and plant‐based diets, which are rich in dietary phytochemicals, may exert beneficial effects on female fertility [28].

Phytochemicals are generally characterised as bioactive, non‐nutritive compounds synthesised as secondary metabolites in plants, predominantly produced as a defence mechanism against environmental stressors, and are widely distributed in fruits, vegetables, whole grains, nuts, and other plant‐derived foods [10]. Key dietary phytochemicals include polyphenols, carotenoids, isoprenoids, phytosterols, saponins, organosulfur compounds, and dietary fibres [10]. These compounds have been shown to confer protective effects against a range of metabolic disorders, including insulin resistance, impaired glucose metabolism, and lipid dysregulation, primarily through their anti‐inflammatory actions and antioxidant properties [29]. In this context, the DPI has been introduced as a novel dietary tool to assess the associations between the consumption of phytochemical‐rich foods and various health outcomes in epidemiologic studies [24].

As this is, to the best of our knowledge, the first study to examine the association between the DPI and DOR, opportunities for direct comparison with previous research are limited. Nonetheless, there is emerging evidence suggesting beneficial effects of adherence to the DPI, as a measure of phytochemical intake, on reproductive disorders such as PCOS [11, 17]. For instance, Chi et al. reported a reverse association between higher adherence to DPI and unfavourable metabolic and hormonal profiles in women with PCOS [17]. Besides, a recent scoping review encompassing 18 clinical trials highlighted the beneficial effects of phytochemical‐rich supplements and diets, which exert antioxidant and anti‐inflammatory properties, leading to improvements in hormonal and metabolic markers among women with PCOS [11], further supporting the potential role of phytochemical intake in reproductive health management. Additionally, both experimental and clinical studies suggest that specific phytochemicals, such as phytoestrogens, flavonoids, and certain herbal extracts, can modulate key reproductive hormones, including LH, FSH, prolactin, oestrogen, and progesterone, particularly in conditions like PCOS and hypogonadism [30, 31]. More specifically, findings from other animal models have demonstrated that various phytochemicals enhance ovarian reserve indicators, particularly AMH levels, suggesting their potential role in preserving follicular function and reproductive capacity [32].

Although human trials on the effects of phytochemicals on ovarian reserve are still lacking, highlighting a key gap in fertility research, several studies have shown that the DPI is inversely associated with a range of health conditions such as metabolic syndrome [33], obesity [34], cancer [35], and chronic kidney disease progression [36]. Notably, in the current study, individuals with higher DPI scores had significantly greater intakes of phytochemical‐rich foods including fruits, vegetables, legumes, whole grains, soy, and tea, further supporting the index's relevance as a marker of health‐promoting dietary patterns and suggesting a potential association underlying our findings. In line with this, previous research has shown that lower fruit intake is associated with prolonged time to pregnancy and an elevated risk of infertility [37]. Accordingly, the Mediterranean Diet, a holistic dietary pattern characterised by high intake of extra virgin olive oil, antioxidant‐ and vitamin‐rich plant foods, whole grains, low‐fat dairy, poultry, and oily fish, has been associated with improved reproductive outcomes. It has been shown that this healthy diet can enhance fertility in both natural conception and assisted reproductive technology (ART) contexts through its anti‐inflammatory and endocrine‐modulating properties [7, 8]. Similarly, Kabodmehri et al. recently reported that adherence to an antioxidant‐rich diet, which is comparable to the DPI and highlights the importance of plant‐based foods and overall antioxidant content, may lower the risk of infertility by alleviating oxidative stress and inflammation [38].

Interestingly, in our study, although higher compliance with DPI scores was linked to lower odds of DOR in the overall population, we did not find a significant association between DPI and AMH or AFC levels among women who were already diagnosed with DOR. As a result, this may suggest that the beneficial effects of phytochemical‐rich diets are more relevant to the prevention or slowing of the decline in ovarian reserve, instead of reversing or improving it once DOR has developed. Another possibility is that, in women with DOR, ovarian function may have fallen to a level where dietary influences no longer exert measurable effects on AMH or AFC. Besides, factors that were not accounted for such as the use of drugs with psychological effects, environmental toxins, stress, hormonal or immune‐related disorders [2], and possible reproductive‐specific genetic factors [39] may have contributed to the lack of significant differences observed. It is also worth noting that among the control group, those in the second DPI quartile had the highest AFC levels compared to other categories, suggesting a possible non‐linear association between DPI and ovarian reserve in healthy women. Consequently, greater longitudinal studies are warranted to better understand the timing and nature of the relationship between phytochemical intake and ovarian function throughout the reproductive lifespan.

Although the exact biological mechanisms relating the DPI to ovarian reserve have yet to be understood, extensive evidence suggests that dietary phytochemicals may help reduce inflammation and oxidative stress [13], both of which may play crucial roles in pathogenesis of reproductive abnormalities [40]. Of note, an imbalance in reactive oxygen species (ROS) levels due to oxidative stress could disrupt the critical reproductive processes including oocyte development, follicular progression, luteal phase regulation, and early placental development, ultimately impacting ovarian function and fertility [40, 41]. In this regard, accumulated evidence has documented the strong anti‐inflammatory properties of phytochemicals [42]. For example, phytochemical compounds such as resveratrol, quercetin, genistein, kaempferol, and daidzein have been reported to reduce inflammation by suppressing pro‐inflammatory cytokines (e.g., interleukins (IL‐6, IL‐1*β*), and tumour necrosis factor‐alpha (TNF‐*α*)) and inhibiting production of nitric oxide through regulation of important signalling pathways like nuclear factor kappa B (NF‐*κ*B) and mitogen‐activated protein kinase (MAPK) [43]. In addition, they have shown to exhibit antioxidant activity by scavenging reactive oxygen and nitrogen species, chelating metal ions, and enhancing the expression of antioxidant enzymes (e.g., catalase, glutathione peroxidase, and superoxide dismutase) [13, 43]. Another potential mechanism may be attributed to the beneficial impacts of these compounds on hormonal regulation which is essential for normal reproductive function [44, 45]. Nonetheless, despite the proposed mechanisms, investigating underlying biological pathways through inflammatory and oxidative stress biomarkers may shed light on how phytochemical‐rich diets influence reproductive capacity.

A key strength of this study is providing new insights into potentially modifiable risk factors, especially overall diet, that may help prevent or manage DOR. Other advantages include matching groups by age and BMI, a large sample size, and accounting for various possible confounders in the analysis. However, this study has several limitations that should be acknowledged. First, causal relationships cannot be established due to the study's retrospective design. Second, the method used to assess DPI is based on the caloric intake of phytochemical‐rich foods, which means it does not accurately reflect total phytochemical intake, as it excludes non‐caloric but phytochemical‐dense items such as spices, green tea, and black tea. Furthermore, this method does not capture the type and quality of phytochemicals consumed. Third, the lack of a region‐specific food composition database for plant‐based nutrients further restricts the precision and generalizability of DPI estimation since phytochemical profiles can vary considerably across regions. Forth, although we adjusted for major confounders, we could not include all possible confounding variables in the statistical models, as adding additional covariates could reduce statistical power. Therefore, the potential influence of unmeasured factors such as psychological stress, environmental toxins, genetic background, endocrine‐related disorders, occupation, or additional lifestyle variables may have affected the validity of the observed association. Fifth, as this study included women seeking care at fertility clinics, our findings may not be generalizable to the general population. This recruitment strategy may introduce selection bias, as women attending fertility clinics could differ in reproductive characteristics from women in the broader community.

In conclusion, the results of our study suggest that adherence to a high phytochemical diet may reduce the chance of having DOR, supporting the significance of the contribution of dietary factors in the prevention and management of reproductive disorders. Whilst further research, particularly of prospective trials, is needed to confirm the present findings. Moreover, to better understand how dietary phytochemical patterns affect ovarian potential, it is essential to elucidate the underlying mechanisms by measurement of inflammatory and oxidative stress markers.

## Author Contributions

Abed Ghavami, Gholamreza Askari and Hatav Ghasemi‐Tehrani; conception and design of the work, Hatav Ghasemi‐Tehrani and Maryam Kalatehjari; study gynecologists, Abed Ghavami, Mahdi Vajdi, Amin Mokari‐Yamchi; analysis and interpretation of data, Maryam Kalatehjari, Abed Ghavami, draft the work and revise it.

## Funding

The present study was supported by a grant from Vice‐Chancellor for Research, Isfahan University of Medical Sciences (Grant No: 2401257).

## Ethics Statement

Written informed consent was obtained from all participants, and the study followed the principles of the Declaration of Helsinki. Ethical approval was obtained from the Ethics Committee of Isfahan University of Medical Sciences (IR.ARI.MUI.REC.1401.297) with support from the Department of Nutrition, Faculty of Medicine.

## Consent

Written informed consent was obtained from all participants.

## Conflicts of Interest

The authors declare no conflicts interest.

## Data Availability

The datasets used and/or analysed during the current study are available from the corresponding author on reasonable request.
